# Investigation of stratum corneum cell morphology and content using novel machine‐learning image analysis

**DOI:** 10.1111/srt.13565

**Published:** 2024-01-26

**Authors:** Takeshi Tohgasaki, Saki Aihara, Mariko Ikeda, Minako Takahashi, Masaya Eto, Riki Kudo, Hiroshi Taira, Ai Kido, Shinya Kondo, Shioji Ishiwatari

**Affiliations:** ^1^ FANCL Research Institute FANCL Corporation Yokohama Kanagawa Japan; ^2^ Software and AI Technology Center Toshiba Digital Solutions Corporation Kawasaki Kanagawa Japan

**Keywords:** artificial intelligence (AI), biomarker, corneocyte cell morphology, dermatology, machine learning image analysis, multivariate analysis, skin condition, stratum corneum

## Abstract

**Background:**

The morphology and content of stratum corneum (SC) cells provide information on the physiological condition of the skin. Although the morphological and biochemical properties of the SC are known, no method is available to fully access and interpret this information. This study aimed to develop a method to comprehensively decode the physiological information of the skin, based on the SC. Therefore, we established a novel image analysis technique based on artificial intelligence (AI) and multivariate analysis to predict skin conditions.

**Materials and Methods:**

SC samples were collected from participants, imaged, and annotated. Nine biomarkers were measured in the samples using enzyme‐linked immunosorbent assay. The data were then used to teach machine‐learning models to recognize individual SC cell regions and estimate the levels of the nine biomarkers from the images. Skin physiological indicators (e.g., skin barrier function, facial analysis, and questionnaires) were measured or obtained from the participants. Multivariate analysis, including biomarker levels ​​and structural parameters of the SC as variables, was used to estimate these physiological indicators.

**Results:**

We established two machine‐learning models. The accuracy of recognition was assessed according to the average intersection over union (0.613), precision (0.953), recall (0.640), and F‐value (0.766). The predicted biomarker levels significantly correlated with the measured levels. Skin physiological indicators and questionnaire answers were predicted with strong correlations and correct answer rates.

**Conclusion:**

Various physiological skin conditions can be predicted from images of the SC using AI models and multivariate analysis. Our method is expected to be useful for dermatological treatment optimization.

## 1 INTRODUCTION

Extensive physiological information is recorded on the stratum corneum (SC). SC cells are formed during the keratinization of cells from the basement membrane to the surface layer. Keratinocytes inherit their innate characteristics during division from epidermal stem cells in the basal layer. With their flattened morphology, they form a flat Kelvin tetradecahedron with tight junctions (TJs) to fill spaces, thereby achieving a regular overlap and arrangement. The TJs and nucleus eventually disappear, revealing the final keratinized structure.^1^, ^2^, ^3^ This process is potentially controlled by skin properties and proteins and is influenced by internal and external conditions. SC cells portray the factors influencing and involved in keratinization. Additionally, SC cell morphology is influenced by the physiological functions of the skin, such as the skin barrier, skin permeability, and nerve elongation.^4^, ^5^, ^6^, ^7^ A relationship between the mechanical and maturation properties of corneocytes and the barrier functions of the SC is suggested.^8^ Therefore, various methods have been proposed for evaluating physiological skin conditions based on the morphological characteristics of keratinocytes and the specific protein content of the SC.

The area of SC cells and the SC exfoliation state have been suggested as indices of the differentiation rate and SC water content, respectively.^9^ Moreover, we previously reported that the expression levels of specific proteins, including heat shock protein 27 (HSP27), macrophage migration inhibitory factor (MIF), interleukin‐1 receptor antagonist (IL‐1Ra), Parkinson's disease 7 (PARK7/DJ‐1), galectin‐7 (GAL‐7), arginase‐1 (ARG1), neutrophil gelatinase‐associated lipocalin (NGAL), fatty acid‐binding protein 5 (FABP5; also known as epidermal FABP), and enolase 1 (Eno‐1), in SC cells are associated with atopic dermatitis and skin barrier properties,^10^, ^11^, ^12^, ^13^, ^14^, ^15^ and that these proteins can be used as biomarkers for evaluating skin conditions. However, the morphological evaluation of SC cells requires visual assessment and recognition, and biomarker measurement in SC cells requires a biochemical assay, such as an immunoblotting or enzyme‐linked immunosorbent assay (ELISA). These approaches require time, additional costs, and sophisticated equipment, which limit the practical application and research of an appropriate evaluation method, necessitating the development of simpler methods. Furthermore, comprehensive studies on the morphological characteristics of SC cells are limited. Hence, improving the evaluation method of the physiological state of the skin based on the morphological characteristics of SC cells has considerable potential.

Advances in machine‐learning technology have significantly increased in recent years, enabling accurate recognition and classification of specific objects in images.^16^, ^17^ This technology has been applied in various fields, such as in the analysis of microscopic and medical images.^18^, ^19^ In the field of dermatology, there have been reports on AI solutions that predict skin moisture content from visible light skin images and skin feature factors.^20^ In this study, we constructed two machine‐learning models; the first model automatically recognized individual SC cells from SC cell images, whereas the second could estimate SC cell biomarker levels related to skin physiology. In addition, we verified the feasibility of estimating skin physiological indicators and skin responsiveness using multiple regression analysis with the numerical output values from these two machine‐learning methods. Our methods may be useful for the optimization of dermatological treatments.

## 2 METHODS

### 2.1 Participants

Data were obtained from two groups of participants. SC cells from 996 healthy Japanese women (20–92 years old; average age, 43.2 years) were collected and used as training and test data to construct the two machine‐learning models. To construct a mathematical model for estimating the physiological parameters of the skin, SC cells were collected, skin physiological parameters were measured (section 2.5), and responses from skin‐related questionnaires were obtained from a different group of 516 healthy Japanese women (20–92 years old; average age, 44.3 years). Written informed consent was obtained from all participants. The study was conducted with the approval of the ethical committee of FANCL Co. Ltd. (approval number: C2020‐035), and the experimental protocols were performed in accordance with the principles of the Declaration of Helsinki.

### 2.2 Collection and imaging of SC cells

SC samples were obtained from the cheeks of the participants via single stripping using skin tape (25 × 25 mm; Horney Layer Checker; Asahi Biomed Co. Ltd, Tokyo, Japan). The collected SC cells were imaged using Dino‐lite AM7515 (AnMo Electronics Corp., New Taipei City, Taiwan) with 8‐bit RGB, 0.41 μm/pixel, and 2592 × 1944 pixels. Images were captured in two to five fields per sample.

### 2.3 Data preparation for machine learning

Two machine‐learning models were constructed: an automatic recognition model of individual SC cell regions and an estimation model of biomarker levels in SC cells. Individual SC cell regions in the images obtained from the 996 participants were visually identified and labeled using the annotation software Labelme (MIT, Cambridge, MA, USA; Figure S1). The annotated image set was expanded via rotation and inversion and then used as training and test images. For the biomarker estimation model for SC cells, 157 samples were used for the measurement and assessment of biomarker values. The model was trained based on the levels of nine biomarkers: HSP27, MIF, IL‐1Ra, DJ‐1, GAL‐7, ARG1, NGAL, FABP5, and Eno‐1, which were quantified using ELISA (section 2.4) and SC cell images.

An instance segmentation model, pre‐learned using ImageNet, was used to construct a machine‐learning model that automatically recognized individual SC cell regions. A convolutional neural network was used for learning and construction of a machine‐learning model for estimating biomarker values from the SC images.

### 2.4 ELISA

Samples were extracted using the T‐PER Tissue Protein Extraction Reagent (Thermo Fisher Scientific, Waltham, MA, USA) or RIPA Lysis and Extraction Buffer (Thermo Fisher Scientific). The concentrations of the nine biomarkers in the extracts were quantified using ELISA. To correct the variable numbers of SC cells collected, the total protein concentrations in the extracts were determined using the Pierce BCA Protein Assay Kit (Thermo Fisher Scientific), and the amount of each biomarker per total protein was calculated. Machine learning was performed using the corrected values.

### 2.5 Skin parameter measurements

Skin parameters were assessed using noninvasive bioengineering measurements. The Tewameter VapoMeter (Keystone Scientific, Tokyo, Japan), SKICON 200EX skin conductance meter (Yayoi Co., Ltd., Tokyo, Japan), and MPA580 Cutometer (Courage + Khazaka Electronic GmbH, Cologne, Germany) were used to determine trans‐epidermal water loss (TEWL), SC water content, and skin elasticity indicators (R0, R2, R5, R6, and R7), respectively. Whole‐facial imaging was used to analyze indicators related to skin elasticity and wrinkles (texture and wrinkle counts), UV damage (UV spots), stains (brown spot count), pores (pore and spot counts), and inflammation (red spot and red vascular counts) using VISIA Evolution System (Canfield Scientific, Fairfield, NY, USA). The room used for the measurements was maintained at a temperature of 21−24°C, with the humidity ranging between 40% and 60%.

### 2.6 Quantification of SC cell shape

For SC cells in each image, individual cell regions were labeled using the constructed automatic cell recognition model. The morphological parameters (area, circumference, roundness, regular polygon approximation, etc.) and intensity values (intracellular brightness mean, standard deviation, etc.) of each labeled cell were quantified. The average and standard deviation of the shape and brightness values between the cells in the image were also calculated. All cell and stratified regions were labeled using the method of Otsu ^21^ for document image binarization, and these areas were quantified (Figure S2).

### 2.7 Questionnaire

The following questions were asked in relation to the responses of the participants’ skin to cosmetics and UV radiation: (1) Cosmetics do not agree with your skin (yes/no); (2) Experienced inflammation and itching caused by cosmetics (very often/often/no); (3) Reddening after sunburn (yes/no); (4) Turned brown after sunburn (yes/no).

### 2.8 Assessment of cell recognition accuracy using the AI model

The consistency of the estimated and annotated regions was confirmed to evaluate the accuracy of the AI model for recognition of the SC cell region constructed in this study. The matching between the predicted and annotated regions in each pixel was classified into true positive (TP), false positive (FP), and false negative (FN), and from these values, intersection over union (IoU), recall, precision, and F‐measure (F‐number) were calculated. The calculation formula for each of these parameters is shown below.

$$Precision\;=\;\frac{TP}{TP+FP}$$

$$Recall\;=\;\frac{TP}{TP+FN}$$

$$F\;=\;\frac{TP}{TP+\frac{1}{2}\left(FP+FN\right)}$$

$$IoU\;=\;\frac{Area\;of\;overlap}{Area\;of\;union}$$

### 2.9 Statistical analysis

Correlation analysis was performed using the Pearson product‐moment correlation test to evaluate biomarker levels predicted using the machine‐learning model. To estimate skin parameters, multiple regression analysis was performed using structural and biomarker values as explanatory variables. The estimation accuracy of each skin parameter via multiple regression analysis was evaluated using correlation analysis. Discriminant analysis was used to predict qualitative variables in the questionnaire responses. The accuracy of this discriminant analysis was assessed via calculating the true/false rate. All statistical analyses were performed using JMP 16.2.0 (SAS Institute Inc., Cary, NC, USA). Results were considered significant at *p* < 0.05.

## 3 RESULTS

### 3.1 Machine‐learning model for automatic recognition of individual SC cell regions

**FIGURE 1:**
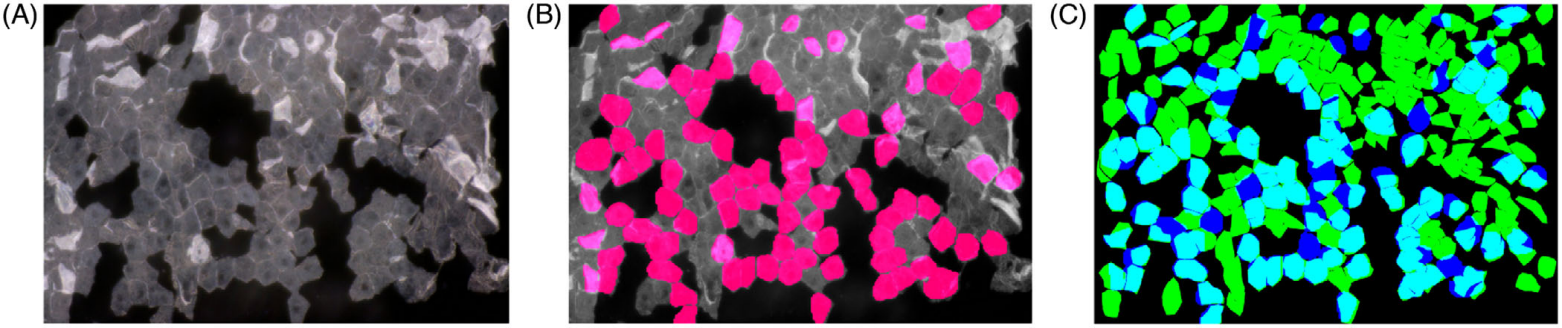
Evaluation of the predicted and annotated regions. Raw image (A). Regions predicted by the machine‐learning model (B), indicated in pink. Merged image (C) showing a green annotated area (false negative), blue predicted area (false positive), and cyan true positive area.

We established two machine‐learning models, one that recognized SC cell regions and another that predicted biomarker protein levels from SC images. Figure 1 shows the quantitative and qualitative evaluations of the predicted and annotated images. The number of cells recognized by the model was lower than that annotated based on human visual judgment. The estimated cell region coincided approximately with the annotated region. Quantitative analysis revealed that the IoU, precision, recall, and F measure (F number) were 0.613, 0.953, 0.640, and 0.766, respectively. Thus, the precision was substantially higher, whereas the recall was lower when the prediction was compared to visual evaluation.

### 3.2 Machine‐learning model for estimating the biomarker levels of SC cells

**FIGURE 2:**
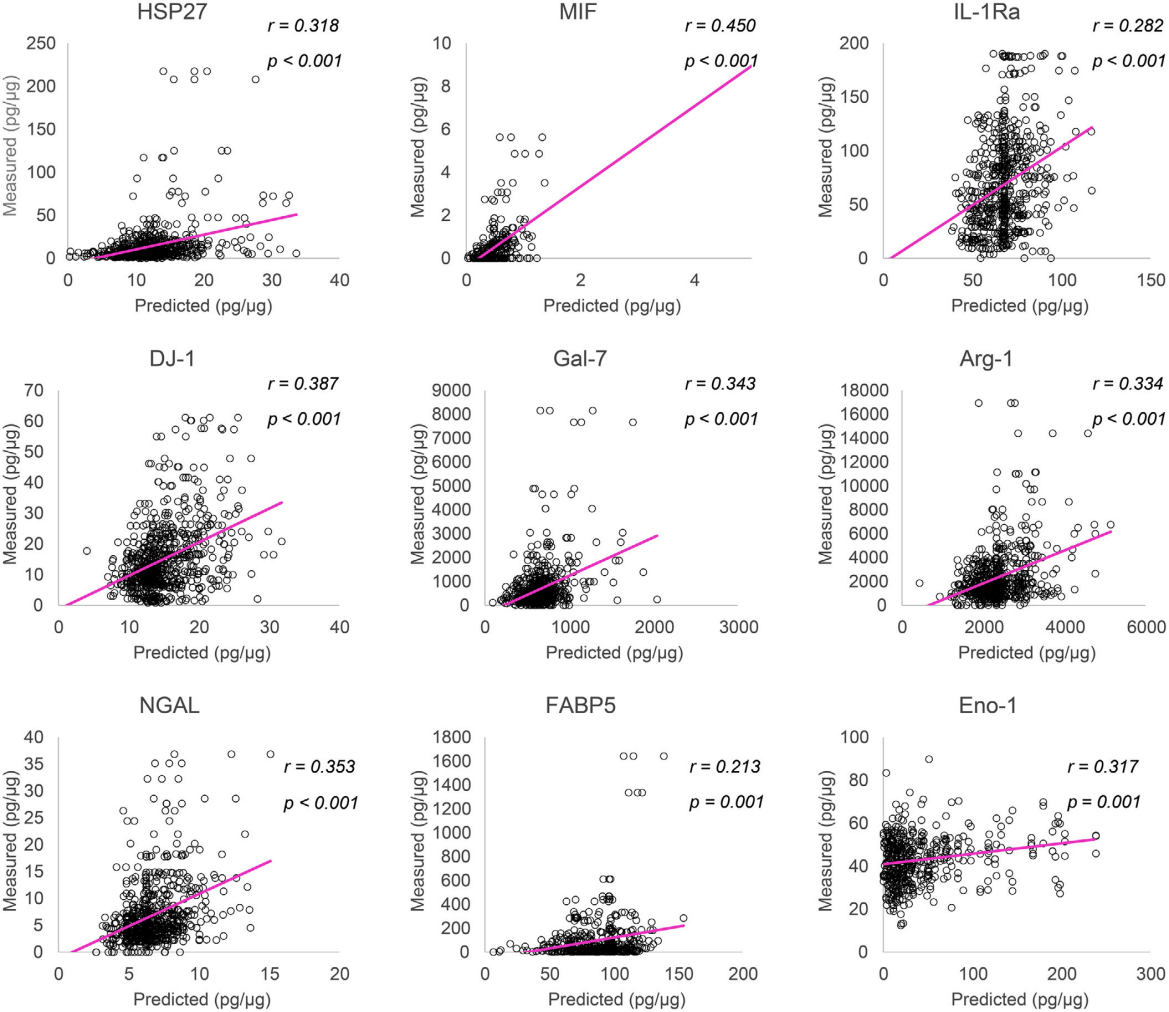
Correlation plots of values predicted by the machine‐learning model ​​and the values measured using ELISA for nine biomarkers in stratum corneum (SC) cells. The *r* values indicate Pearson's correlation coefficients.

The results of the correlation analysis showed that the predicted values of the nine biomarkers were significantly correlated with the measured values (*r* = 0.213–0.450, *p* < 0.001; Figure 2). In the comparison between the estimated and measured values, there were minor differences in the median; however, the estimated values tended to be smaller in the maximum range than the measured values, indicating that the AI model tended to output estimated values within a narrower range, compared to the actual measurements. For example, HSP27 (median of estimated: 12.00, measured: 8.04; maximum of estimated: 33.67, measured: 217.48), MIF (median of estimated: 0.46, measured: 0.16; maximum of estimated: 1.37, measured: 5.63), and IL‐1Ra (median of estimated: 68.05, measured: 60.00; maximum of estimated: 117.22, measured: 190.23).

### 3.3 Estimating skin physiological indicators from SC images

**FIGURE 3:**
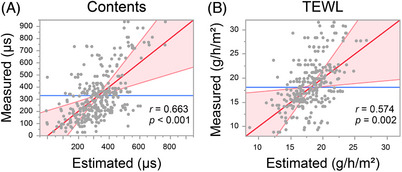
Correlation plots of values predicted by the machine‐learning model and measured values of SC water content (A) and trans‐epidermal water loss (TEWL) (B). The *r* values indicate Pearson's correlation coefficients.

**FIGURE 4:**
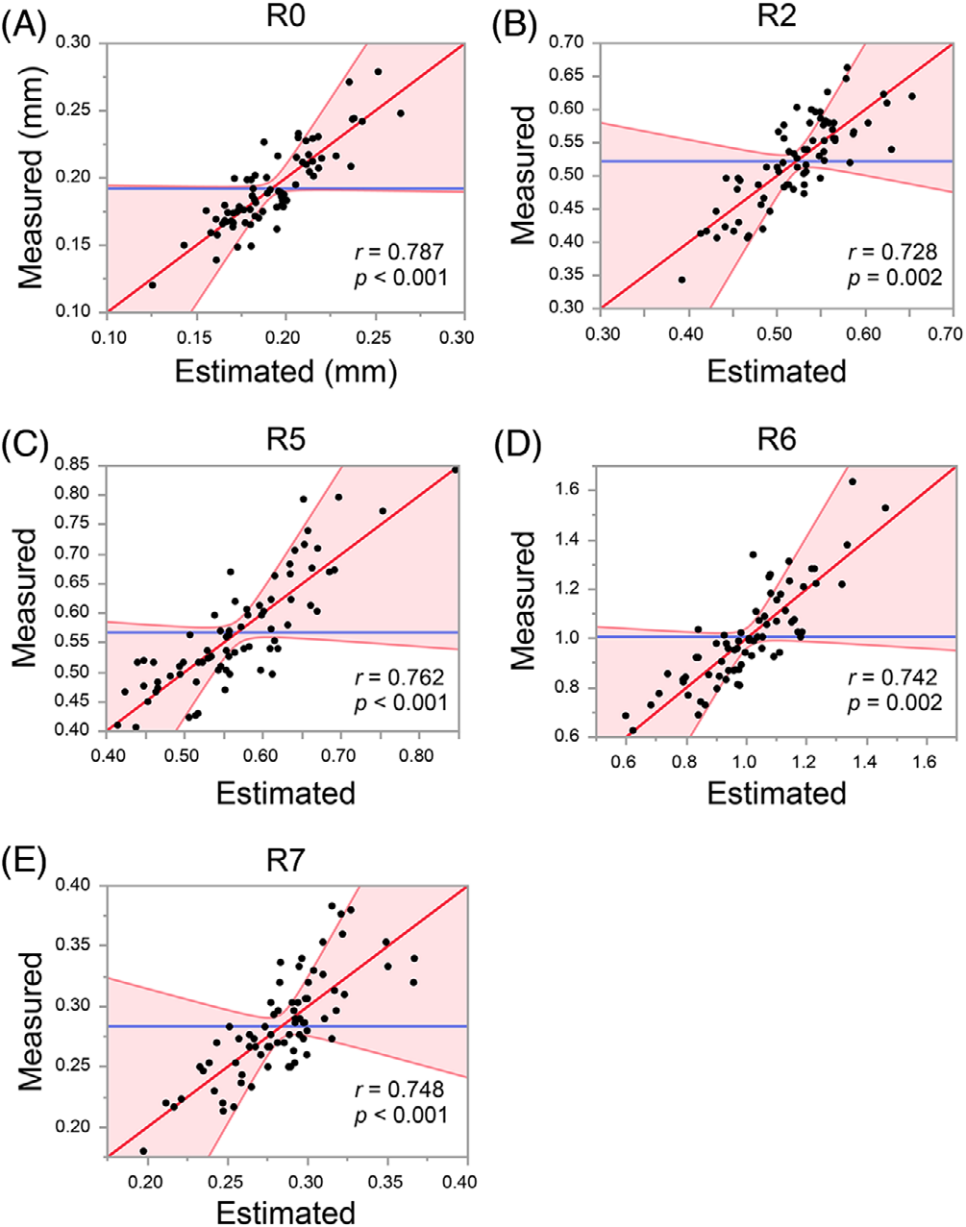
Correlation plots of values predicted by the machine‐learning model and measured values of various elasticity indicators, R0 (A), R2 (B), R5 (C), R6 (D), and R7 (E). The *r* values indicate Pearson's correlation coefficients.

**FIGURE 5:**
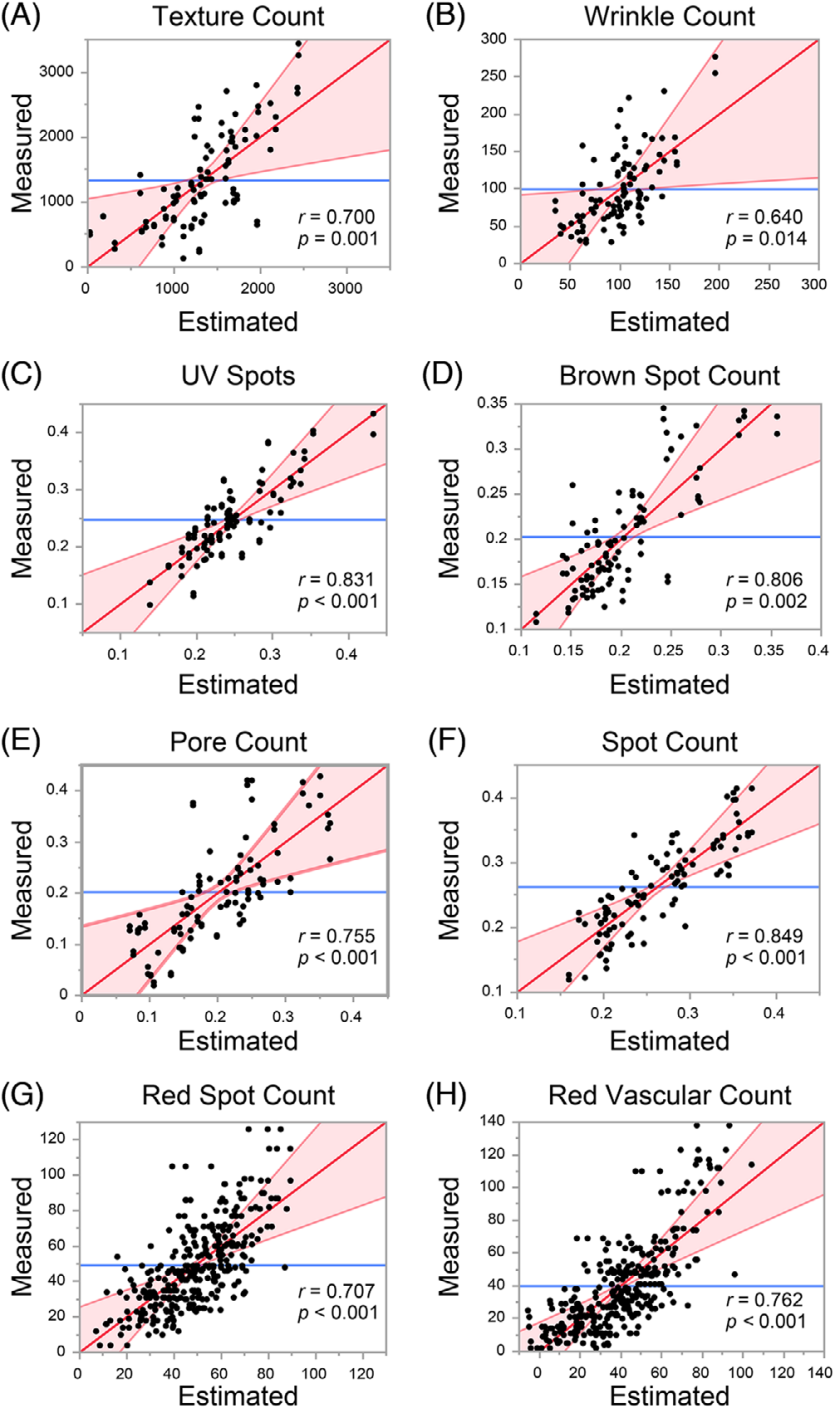
Correlation plots of values predicted by the machine‐learning model and measured values of various skin parameters: texture count (A), wrinkle count (B), UV spots (C), brown spot count (D), pore count (E), spot count (F), red spot count (G), and red vascular count (H). Counts were measured from whole‐face imaging using VISIA. The *r* values indicate Pearson's correlation coefficients.

We then investigated the feasibility of estimating skin physiological indicators via instrumental measurement using images of SC cells. The measured values of SC water content and TEWL were significantly correlated (SC water content: *r* = 0.663, *p* < 0.001, TEWL: *r* = 0.574, *p* = 0.002) with the estimated values from the multiple regression analysis (Figure 3). In particular, IL‐1Ra, GAL‐7, and ARG‐1 levels and elliptic approximation significantly contributed to the estimation of SC water content, whereas HSP27, GAL‐7, and DJ‐1 levels and cell circumference significantly contributed to the estimation of TEWL. The values of the elasticity indicators, R0, R2, R5, R6, and R7, showed a significantly strong correlation with the estimated values (Figure 4). HSP27 and DJ‐1 levels, average intracellular intensity value, and average long‐short side ratio of the rectangle circumscribing a cell were found to have high common contributions to these parameters. Facial indicators (texture, wrinkle, UV spot, brown spot, pore, spot, red spot, and red vascular counts) were obtained from the whole‐face imaging device VISIA, and a significant and strong correlation with the estimated values was shown for each index (Figure 5). The GAL‐7 and ARG‐1 levels, average long‐short side ratio of the rectangle circumscribing a cell, and average intracellular intensity values were strongly correlated with indicators of elasticity and wrinkles. The MIF and DJ‐1 levels and number of angles of a regular polygon to be approximate (standard deviation) were strongly correlated with the UV damage index. The HSP27 and MIF levels, number of angles of a regular polygon to be approximate (average), and average intracellular intensity value were strongly correlated with brown spots. NGAL, ARG‐1, and the average cell circumference were closely associated with the pore index. HSP27, MIF, and the average area of the rectangle circumscribing the cell were strongly correlated with each index related to the spots. IL‐1Ra, DJ‐1, and the number of angles of a regular polygon to be approximate (average) were strongly correlated with the indicators of inflammation. The top five contributing parameters for predicting each indicator, with the associated *t*‐value, are shown in Table S1.

### 3.4 Estimating skin responsiveness from SC images

**FIGURE 6:**
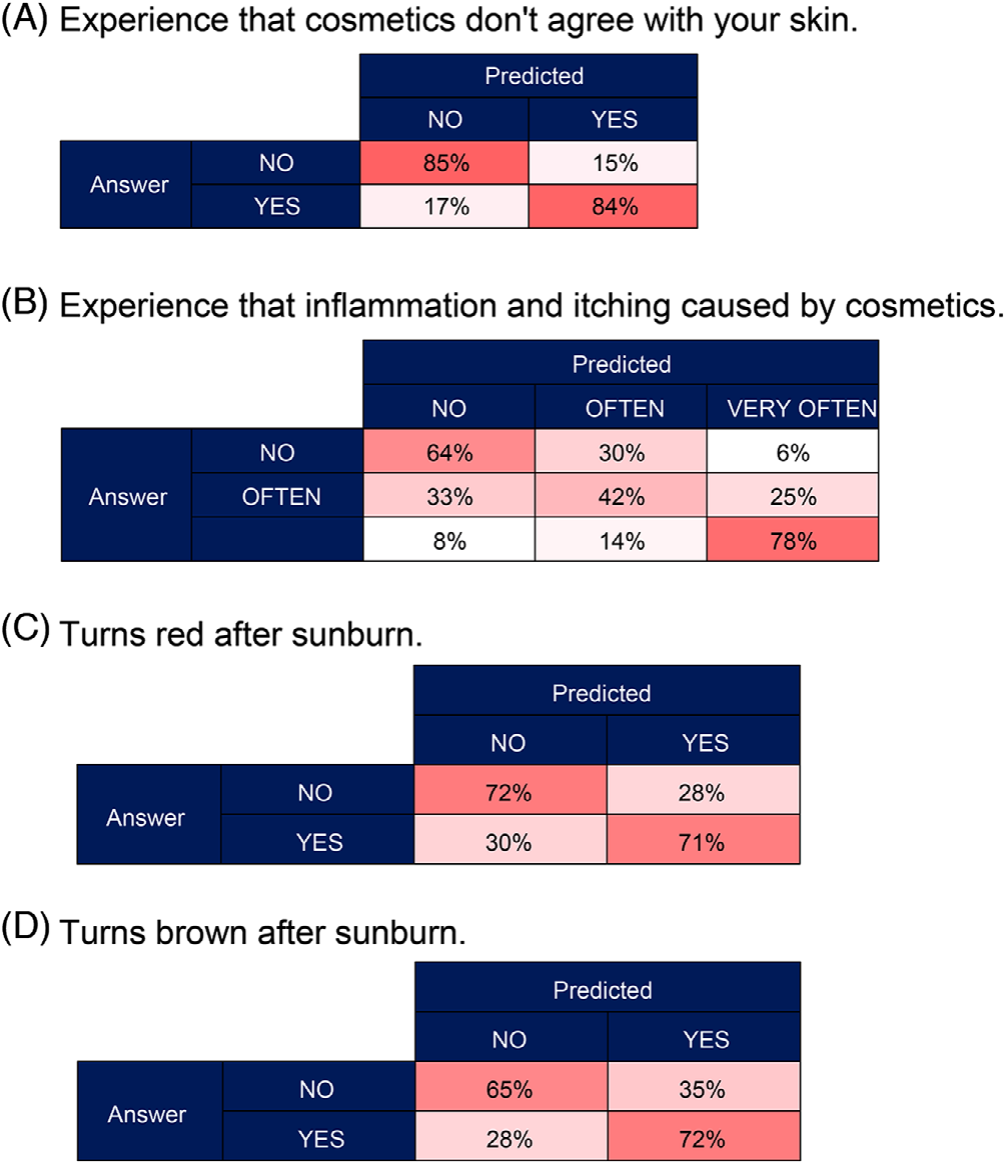
Distribution ratios of the predictions and the actual answers from the skin condition questionnaire.

Figure 6 shows the distribution ratios of the estimated and actual answers. The estimation of the cosmetics suitability with the participants’ skin was achieved, with a correct answer rate of 84.3%. The overall correct answer rate for experiencing inflammation or itching caused by cosmetics was 57.9%, which was relatively low. However, for the answer “very often,” the correct answer rate was 78.3%, and when classified by “experienced or not,” the correct answer rate was 72.4%. For answers indicating the skin turning red or brown due to UV exposure, the correct answer rates were 70.9% and 68.5%, respectively.

## 4 DISCUSSION

In this study, we developed two machine‐learning models, one that automatically recognized individual SC cell regions in an image and another that predicted the levels of biomarkers in the SC from the image. The SC cell regions were recognized using the first model with a substantially high precision accuracy, despite low IoU and recall; however, as the IoU was calculated via including the area of the cells that the model did not recognize, the SC cells detected using this model were recognized at a higher IoU. To evaluate the individual morphological characteristics of SC cells, the accurate determination of the morphology of typical cells is necessary, even in small numbers. Therefore, the precision and IoU values of a model have a higher priority than its recall. The model constructed in this study can recognize the morphological features of SCs. Visual judgment and annotation usually require approximately 1 h per SC cell image, whereas the machine‐learning model can complete this task within a few seconds. Therefore, this method is useful for real‐time skin and high throughput evaluations.

In this study, the model for estimating biomarker levels roughly predicted the measured levels quantified using ELISA. The smaller estimation of biomarker levels and narrow distributions using the model may indicate that the training data were biased in terms of median values, highlighting the necessity of training the model with uniformly distributed data. Measuring biomarkers in the SC requires extensive time, funds, and specialized equipment. Moreover, there are limitations to the types of biomarkers that can be measured from a single sample. Our machine‐learning model can easily and quickly determine the approximate number of multiple markers without these limitations. However, further improvements in the estimation accuracy are necessary via increasing training data, optimizing the imaging device and settings, and optimizing the module in machine learning. A previous study reported that a combination of a visible‐light skin image and skin feature factors ​​improves the water content prediction accuracy.^20^ Therefore, multiple regression analysis with the predicted values and SC cell morphological parameters also has the potential to improve estimation accuracy.

Using the morphological parameters and biomarker levels of the SC cells from the two machine‐learning models, verification of the physiological condition of the skin was performed using multiple regression analysis and discriminant analysis.

The relationship between SC characteristics and the physiological state of the skin has been extensively studied, and some methods have been developed for use in clinical settings.^9^, ^10^, ^11^, ^12^, ^13^, ^14^, ^15^ The two machine‐learning models developed in this study for imaging SC cells offer a convenient approach to obtain SC cell morphological parameters and estimate the levels of nine biomarkers. Thus, we were able to quantify the characteristics of SC cells comprehensively and quickly, which can progress research toward understanding the relationship between the characteristics of SC cells and the physiological conditions of the skin. In addition, we clarified the relationship of SC cells with several physiological indicators; however, many other physiological indicators and future skin conditions can be potentially estimated, and the effects of cosmetics can be predicted via analyzing SC cells. Furthermore, multiple regression analysis can be used to identify key biomarkers or morphological parameters that are strongly correlated with each skin physiological state. These parameters are useful for elucidating the mechanisms underlying skin physiology. For example, relationships between HSP27 and GAL‐7 in SC cells and barrier indicators, SC water content, and TEWL have been reported.^13^, ^15^ Our data also suggest that these relationships are stronger than those of other parameters. Conversely, we found that the non‐uniformity of the intensity of the SC cells, which has scarcely been studied, is highly related to the pore index. We identified several relationships, which may provide key information for the discovery of new dermatological mechanisms. In conclusion, our image analysis technique can help interpret the enormous amount of information found in SC cells.

## CONFLICT OF INTEREST STATEMENT

The authors have no conflict of interest to declare.

## ETHICS STATEMENT

The study was conducted with the approval of the ethical committee of FANCL Co. Ltd. (approval number C2020‐035) and the experimental protocols were performed in accordance with the principles of the Declaration of Helsinki.

## PATIENT CONSENT

Written informed consent was obtained from all participants.

## Supporting information

Supporting Information

## Data Availability

None.
