# Supplementary material for: Investigation of stratum corneum cell morphology and content using novel machine‐learning image analysis
Source: Skin Res Technol. 2024 Jan 26;30(2):e13565. doi: 10.1111/srt.13565 (PMC10818130; doi:10.1111/srt.13565)
Supplement: Supplementary file 1 — Supporting Information [file SRT-30-e13565-s001.docx]

**Supporting Information**

**
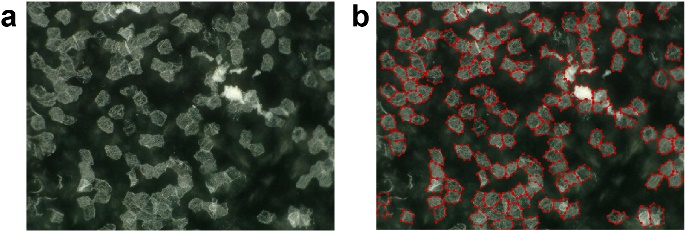
**

**Supplementary Figure S1**. Annotation of stratum corneum (SC) cells from images. Raw image (a) and annotated data (b), where the red frame indicates the SC region recognized by human visualization.


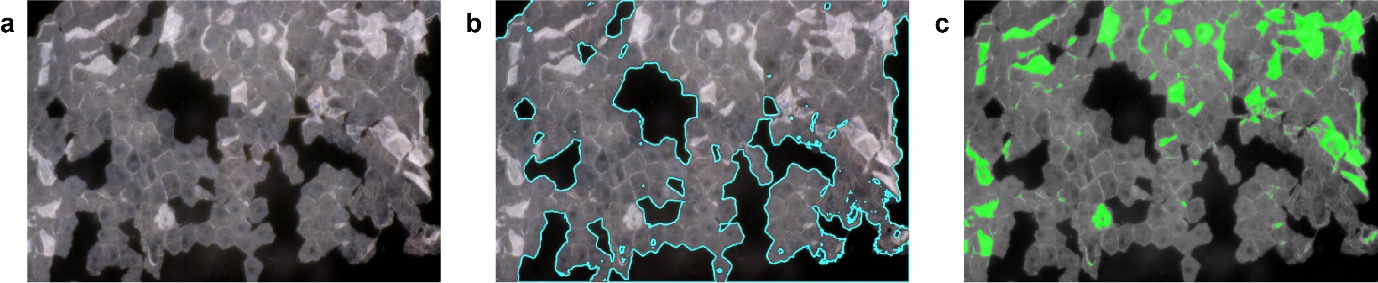


**Supplementary Figure S2**. Identification of the stratum corneum (SC) cell regions and multi-layered regions via binarization. Raw image (a). All SC cell regions (b) and stratified regions (c) were labeled using the method described by Otsu^1^. The blue line indicates the boundary between the cell area and the background, and the green area indicates the stratified regions.

**Supplementary Table S1**. Top five highest contributors and *t*-value to stratum corneum (SC) water content and trans-epidermal water loss (TEWL); various elasticity indicators and *t*-value; and various skin parameters measured from whole-face imaging, VISIA, and *t*-value.

| **SC water content** | |
| --- | --- |
| **Parameter** | ***t-value*** |
| IL-1RA | -2.89 |
| Arg1 | 2.49 |
| elliptic approximation | 2.24 |
| Gal7 | -2.14 |
| NGAL | 1.11 |
| **TEWL** | |
| **Parameter** | ***t-vale*** |
| HSP27 | -2.95 |
| Gal7 | 2.94 |
| cell circumference (average) | -2.85 |
| DJ-1 | -2.65 |
| IL-1Ra | -2.38 |
| **R0** | |
| **Parameter** | ***t-*value** |
| Gal7 | 4.73 |
| Long-short side ratio of a rectangle circumscribing a cell (average) | -4.27 |
| Intracellular intensity value (average) | -3.44 |
| Roundness (standard deviation) | 3.4 |
| Standard deviation of intracellular intensity value (average) | 3.24 |
| **R2** | |
| **Parameter** | ***t*-value** |
| Intracellular intensity value (average) | 3.66 |
| Roundness (standard deviation) | -3.03 |
| Long-short side ratio of a rectangle circumscribing a cell (average) | 3 |
| Cell circumference (standard deviation) | 2.88 |
| HSP27 | 2.76 |
| **R5** | |
| **Parameter** | ***t-*value** |
| Intracellular intensity value (average) | 4.37 |
| MIF | -3.31 |
| HSP27 | 2.99 |
| Standard deviation of intracellular intensity value (average) | -2.68 |
| Long-short side ratio of a rectangle circumscribing a cell (average) | 2.54 |
| **R6** | |
| **Parameter** | ***t*** |
| cell circumference (average) | 3.79 |
| DJ-1 | -3.51 |
| Cell area　(average) | -2.24 |
| Roundness (average) | 2.15 |
| Standard deviation of intracellular intensity value (average) | -2.09 |
| **R7** | |
| **Parameter** | ***t-*value** |
| Intracellular intensity value (average) | 4.05 |
| Cell circumference (standard deviation) | 3.47 |
| HSP27 | 3.45 |
| MIF | -2.74 |
| Number of angles of a regular polygon to approximate (average) | 2.74 |
| **Texture Count** | |
| **Parameter** | ***t-*value** |
| Long-short side ratio of a rectangle circumscribing a cell (average) | -0.91 |
| Intracellular brightness value (average) | -2.54 |
| Gal7 | 2.22 |
| Arg1 | 2.92 |
| Cell circumference (standard deviation) | 0.98 |
| **Wrinkle Count** | |
| **Parameter** | ***t-*value** |
| Long-short side ratio of a rectangle circumscribing a cell (average) | -3.2 |
| Intracellular brightness value (average) | -2.91 |
| Gal7 | 2.43 |
| Arg1 | 2.01 |
| Cell circumference (standard deviation) | 2.01 |
| **UV Spots** | |
| **Parameter** | ***t-*value** |
| Number of angles of a regular polygon to approximate (standard deviation) | 5.08 |
| Intracellular brightness value (standard deviation) | -4.63 |
| Number of angles of a regular polygon to approximate (average) | -4.58 |
| Intracellular brightness value (average) | -3.56 |
| HSP27 | -3.17 |
| **Brown Spot Count** | |
| **Parameter** | ***t-*value** |
| MIF | 4.79 |
| HSP27 | -4.46 |
| NGAL | -3.97 |
| Number of angles of a regular polygon to approximate (average) | -3.88 |
| Intracellular brightness value (average) | -3.69 |
| **Pore Count** | |
| **Parameter** | ***t-*value** |
| NGAL | 3.97 |
| cell circumference (average) | 3.63 |
| Arg1 | 2.9 |
| Cell area　(average) | -2.74 |
| Cell circumference (standard deviation) | 2.52 |
| **Spot Count** | |
| **Parameter** | ***t-*value** |
| Area of ​​the rectangle circumscribing the cell　(average) | -4.44 |
| MIF | 3.72 |
| HSP27 | -3.56 |
| cell circumference (average) | 3.12 |
| Gal7 | 3.09 |
| **Red spot Count** | |
| **Parameter** | ***t-*value** |
| IL-1Ra | -2.65 |
| Arg1 | 2.45 |
| DJ-1 | -2.35 |
| Number of angles of a regular polygon to approximate (average) | -2.14 |
| cell circumference (average) | -1.88 |
| **Red Vascular count** | |
| **Parameter** | ***t-*value** |
| Hsp27 | 5.08 |
| IL-1Ra | -4.63 |
| Number of angles of a regular polygon to approximate (average) | -4.58 |
| DJ-1 | -3.56 |
| Standard deviation of intracellular brightness value (standard deviation) | -3.17 |

**References**

1. Otsu N. A threshold selection method from gray-level histograms. IEEE Transactions on Systems, Man, and Cybernetics. 1979;9(1):62-66. doi:10.1109/tsmc.1979.4310076
